# Supplementary material for: Rapid turnover of life-cycle-related genes in the brown algae
Source: Genome Biol. 2019 Feb 14;20:35. doi: 10.1186/s13059-019-1630-6 (PMC6374913; doi:10.1186/s13059-019-1630-6)
Supplement: Supplementary file 3 — Protein alignments, corresponding Gblocks html file and CDS sequences for the lineage conserved gametophyte-biased genes. (ZIP 2109 kb) [file 13059_2019_1630_MOESM3_ESM.zip › Con_GA_alignments/Ec-27_006340.aa_ali.fasta-gb.htm]

Ec-27\_006340.aa.fasta.trans.aa\_ali.fasta


## Gblocks 0.91b Results

Processed file: **Ec-27\_006340.aa.fasta.trans.aa\_ali.fasta**  
Number of sequences: **2**  
Alignment assumed to be: **Protein**  
New number of positions: **370** (selected positions are underlined in blue)

```
                         10        20        30        40        50        60
                 =========+=========+=========+=========+=========+=========+
Ec-27_006340     MPHTNRARRADHYRSIDGAGSSGNNSSSATTSFNRNHGGGRKRSHQEQDGGALDYNHDTN
MSTRG.49489.1    MPHTNRARRADHYRSIDGKGTASGSSNPSTATSN-NSSSRRHSHHDHQDSSALNSSTRGG
                 ################################## ##################       


                         70        80        90       100       110       120
                 =========+=========+=========+=========+=========+=========+
Ec-27_006340     -VKASERYGGRSPGAGGGGGGGWGAGRGRGGARSGGGGTGRGGGGGRGGAAEEAGGGSGG
MSTRG.49489.1    GPSSSERHGGRSAGRGR--GG-----RGPGGGRQTANGRGMGRGGGSGRPSETDG-----
                     ############          #############################     


                        130       140       150       160       170       180
                 =========+=========+=========+=========+=========+=========+
Ec-27_006340     IAGSSTAAGRGKGKGKNPSAKNRIRSLTRLMNKPGIEEEQRLHLQRQVEELEGKVSEREK
MSTRG.49489.1    --VDPPPAGGSKGKSKNPSVKNRIRGLTRLMNKPGIEEEQRLHLQQQVAELEGRLSEREK
                        #####################################################


                        190       200       210       220       230       240
                 =========+=========+=========+=========+=========+=========+
Ec-27_006340     QERERTLAIKYHKAKFFDRRKALRRLAQIHRKLKGDIEEAARAELERKRDTEEADLKYVL
MSTRG.49489.1    HERERALAIKYHKAKFFDRRKALRRLAQIHRKLKGDIEESARAELEQKRESEEADLKYVL
                 ############################################################


                        250       260       270       280       290       300
                 =========+=========+=========+=========+=========+=========+
Ec-27_006340     YFPKDKKYLPLGKGKEGPPRAQRSHVRFLKEALVRGEAAGWTEFQENLDKFVTGGGEGGR
MSTRG.49489.1    YFPKDKKYLPLGKGKEGPPRAQRSHERFLKEAFSRGEAAGWTEFQENLDKFVTNGGGTGT
                 ########################################################### 


                        310       320       330       340       350       360
                 =========+=========+=========+=========+=========+=========+
Ec-27_006340     PKVAVDAAAAAEEG---------DVKRNK-GIGALPVVAQQKLWDDGEDNSSAEGEGTGK
MSTRG.49489.1    GRARMEPSPPPDAGGDARLGNGRGLKRKKVGLDALPVVAQEKLWDDGGGDASNSSNGQET
                                               ##############################


                        370       380       390       400       410       420
                 =========+=========+=========+=========+=========+=========+
Ec-27_006340     SDAEESESDSG-DGVAGQQGAGKDAAPAARGKKVKRQPTESGSGSWEPEQGEAAGDSSDS
MSTRG.49489.1    VGAGTEESDSEEDASAARSRTEKGKKQLLRGQQE-GSALEEASG----SEGGAGEDTSDS
                 ##########  ###################                   ##########


                        430       440       450       460       470       480
                 =========+=========+=========+=========+=========+=========+
Ec-27_006340     AGELSGDEGFGAAASDEDDAYSFAEARMRWASGGGGSTSGRSDGSDTDGHDSDGSVGDIY
MSTRG.49489.1    EGEISGDEGFGAAASDEEDAYSFAEARMKWASGGGG------------------------
                 ####################################                        


                        490       500       510       520       530       540
                 =========+=========+=========+=========+=========+=========+
Ec-27_006340     ADALARSNGGGDVTREDAPAGKTVVAAGGGAVDGVTSRVDGGVNGGEGGLLLVDGARDED
MSTRG.49489.1    -------------------AAKSAAAAAAT------------------------------
                                                                             


                        550       560       570       580       590
                 =========+=========+=========+=========+=========+====
Ec-27_006340     SPRATKDPRVKRTQQAPGGHGYRTGVAAGEGGEGLQAPEGPHLAAEAEGEGEEQ
MSTRG.49489.1    ----------------------------------------------------AA
```

```
Parameters used
Minimum Number Of Sequences For A Conserved Position: 2
Minimum Number Of Sequences For A Flanking Position: 2
Maximum Number Of Contiguous Nonconserved Positions: 8
Minimum Length Of A Block: 10
Allowed Gap Positions: None
Use Similarity Matrices: Yes
```

```
Flank positions of the 8 selected block(s)
Flanks: [1  34]  [36  53]  [65  76]  [87  115]  [128  299]  [331  370]  [373  391]  [411  456]  

New number of positions in Ec-27_006340.aa.fasta.trans.aa_ali.fasta-gb:  370  (62% of the original 594 positions)
```
